# Mesenchymal stem cells and Interleukin-6 attenuate liver fibrosis in mice

**DOI:** 10.1186/1479-5876-11-78

**Published:** 2013-03-26

**Authors:** Ghazanfar Ali Nasir, Sadia Mohsin, Mohsin Khan, Sulaiman Shams, Gibran Ali, Shaheen N Khan, Sheikh Riazuddin

**Affiliations:** 1 National Center of Excellence in Molecular Biology, University of the Punjab, 87-West Canal Bank Road, Lahore, Pakistan

**Keywords:** Mesenchymal stem cells, Liver fibrosis, Hepatocytes, Interleukin-6

## Abstract

**Background:**

Mesenchymal stem cell (MSC) transplantation has emerged as a promising therapy for liver fibrosis. Issues concerning poor MSC survival and engraftment in the fibrotic liver still persist and warrant development of a strategy to increase MSC potency for liver repair. The present study was designed to examine a synergistic role for Interleukin-6 (IL-6) and MSCs therapy in the recovery of carbon tetrachloride (CCl_4_) induced injured hepatocytes in vitro and in vivo.

**Methods:**

Injury was induced through 3 mM and 5 mM CCl_4_ treatment of cultured hepatocytes while fibrotic mouse model was established by injecting 0.5 ml/kg CCl_4_ followed by treatment with IL-6 and MSCs_._ Effect of MSCs and IL-6 treatment on injured hepatocytes was determined by lactate dehydrogenase release, RT-PCR for (*Bax, Bcl-xl, Caspase3, Cytokeratin 8, NFκB, TNF-α*) and annexin V apoptotic detection. Analysis of MSC and IL-6 treatment on liver fibrosis was measured by histopathology, PAS, TUNEL and Sirius red staining, RT-PCR, and liver function tests for Bilirubin and Alkaline Phosphatase (ALP).

**Results:**

A significant reduction in LDH release and apoptosis was observed in hepatocytes treated with a combination of MSCs and IL-6 concomitant with upregulation of anti-apoptotic gene *Bcl-xl* expression and down regulation of *bax, caspase3, NFκB* and *TNF-α*. Adoptive transfer of MSCs in fibrotic liver pretreated with IL-6 resulted increased MSCs homing and reduced fibrosis and apoptosis. Hepatic functional assessment demonstrated reduced serum levels of Bilirubin and ALP.

**Conclusion:**

Pretreatment of fibrotic liver with IL-6 improves hepatic microenvironment and primes it for MSC transplantation leading to enhanced reduction of liver injury after fibrosis. Synergistic effect of IL-6 and MSCs seems a favored therapeutic option in attenuation of liver apoptosis and fibrosis accompanied by improved liver function.

## Background

Mesenchymal stem cells (MSCs) are multipotent adult stem cells present in bone marrow, adipose tissue and cord blood and have emerged recently as an attractive candidate for liver repair. MSCs have been shown to form functional hepatocytes in vitro [1,2] and possess the ability to secrete soluble factors stimulating endogenous parenchymal cells to support tissue recovery [3,4]. Moreover, MSC transplantation in a liver fibrosis model can reduce fibrosis [5] and restore depleted hepatic function [5-7]. Similarly, treatment with bone marrow derived stem cells can attenuate liver fibrosis by preservation of metalloproteinase levels in fibrotic liver [8].

During the course of liver fibrosis different cytokines, chemokines and growth factors are released as part of the inflammatory response. Among these secreted factors, IL-6 is a pleiotropic cytokine involved in inflammatory pathways, hematopoiesis and immune regulation. Recent evidence implicates IL-6 in survival and regeneration of hepatocytes through Nuclear Factor-kappa B (*NF-κB*) signal transduction and the Ras–MAPK (mitogen-activated protein kinase) pathway [9]. Increased liver injury was observed on CCl_4_ induction in IL-6−/− mice due to *TNF-α* production showing the role of IL-6 downstream of *TNF-α* in hepatoprotection [10]. Hepatocyte apoptosis is attenuated through IL-6 and many of the antiapoptotic genes like *Bcl-xl*, *Bcl-2* and FLIP are upregulated [11]. Exogenous IL-6 treatment corrected the defects in cell proliferation in IL-6−/− mice showing its role as mitogenic agent in liver regeneration after partial hepatectomy [12,13]. In addition, positive effects of IL-6 on proliferation and DNA synthesis have been observed on primary hepatocyte cultures [14].

Accumulating evidence suggests that MSCs can home and migrate to injured liver but are unable to differentiate into hepatocytes rather promoting fibrogenesis in vivo [15,16]. Moreover, issues with MSC engraftment and long term survival within hostile liver microenvironment may also adversely affect the outcome of MSC therapy for liver repair. The aim of the present study was to enhance MSC potential for hepatic repair after CCl_4_ induced liver injury in mouse. We hypothesized that priming hepatic microenvironment with IL-6 would allow an increase in MSC mediated regenerative response by imparting protection to existing hepatocytes. We demonstrate that IL-6 and MSCs synergistically enhance hepatic repair, reduce liver fibrosis and improve overall hepatic function compared to either of the treatments alone.

## Materials and methods

### Animals

The investigation conforms to the *Guide for the Care and Use of Laboratory Animals* published by the US National Institutes of Health (NIH Publication No. 85–23, revised 1985). All animals were treated according to procedures approved by the Institutional Review Board (IRB) at the National Center of Excellence in Molecular Biology, Lahore, Pakistan.

### Cell culture

Mesenchymal stem cells (MSCs) were isolated from tibias and femora of 2 months old C57BL/6 mice (n=10) according to their ability to adhere to plastic surface of a culture flask and were cultured as described previously [17].

### Hepatocyte isolation

Hepatocytes were isolated from C57BL/6 mice (n=20) according to the two step perfusion method as described previously [18]. Isolated hepatocytes were plated at a concentration of 1 x 10^4^ cells/cm^2^ in collagen coated plates (Becton Dickinson, USA) in RPMI 1640 medium (Sigma Aldrich, USA) supplemented with 100 ug/ml streptomycin (MP Biomedicals, USA), 100 units/ml penicillin (MP Biomedicals, USA) and 10% fetal bovine serum (Sigma Aldrich, USA) in a humidified incubator at 5% CO_2_ and 37°C temperature. Medium was replaced after 3 and 24 hrs after seeding followed by various treatments.

### In vitro co-culture model

Hepatocytes were plated on a 6-well collagen coated plate (Becton Dickinson, USA) at a concentration of 1 × 10^4^ cells/cm^2^ and were subjected to injury with 3 mM and 5 mM Carbon tetrachloride (CCl_4_, Merck, Germany) dissolved in DMSO (Merck, Germany) [19,20]. Injured hepatocytes were treated with Interleukin-6 [(IL-6, 10 ng/ml) (Sigma Aldrich, USA)] for 24 hours followed by MSCs administration in a transwell culture system with Dulbecco's Modified Eagle Medium (DMEM) (sigma) medium having 10% FBS, 100 U/ml penicillin and 100 μg/ml streptomycin. Hepatocyctes were divided into 5 groups as, non-treated, CCl_4_, IL-6, MSCs and MSCs + IL-6 treated hepatocytes. Co-culture lasted for 24 hours and hepatocytes were harvested for RNA extraction, apoptosis analysis and LDH cytotoxicity tests [21].

### LDH Assay

Cytotoxicity was analyzed through Lactate dehydrogenase assay according to manufacturer’s protocol (Sigma Aldrich, USA) in hepatocytes co-cultured with MSCs in the presence or absence of IL-6. Normal, CCl_4_ and IL-6 treated hepatocytes were used as controls. Assay was run in triplicate for each sample and absorbance was measured at 490nm [22].

### Apoptosis

FITC-Annexin V kit (Abcam, USA) was used for detection of apoptosis in all of the above mentioned in vitro groups 24 hours after co-culture. Cells were incubated with Annexin-V for 15 minutes, fixed in 2% paraformaldehyde and stained with DAPI (MP Biomedicals, USA).

Apoptosis was estimated on frozen liver sections of control and treatment groups by TUNEL assay kit (Millipore, USA). Sections were fixed by 4% Paraformaldehyde and stained with TUNEL assay kit according to manufacturer’s protocol. Three sections were selected for each mouse and three mice per treatment.

### CCl_4_ induced fibrotic liver model

Female C57BL/6 mice (6–8 weeks old) were subjected to liver fibrosis by injecting 1 ml/kg CCl_4_ (Merck, Germany) in olive oil (1:1) intraperitoneally for four weeks as previously described [23].

### In vivo IL-6 treatment

IL-6 (Sigma Aldrich, USA) was administered at a dose of 100μg/kg intraperitonealy 24 and 48 hours after completion of 4 week CCl_4_ treatment. Mice were divided into five groups i.e. non-treated, CCl_4_, IL-6, MSCs and MSCs + IL-6 treated mice with n=10 animals in each group.

### MSC Transplantation

MSCs were labeled with PKH-26 fluorescent cell linker dye (Sigma Aldrich, USA) for their detection in the liver post transplantation as shown previously [23]. MSCs + IL-6 groups received MSC transplantation 4 hours after completion of IL-6 injections. Anesthesia was induced and abdomen was neatly cut below diaphragm. After exposing liver, approximately 10^6^ cells/ml were transplanted directly in to liver lobes at three lateral and two median sites with a 30 G syringe.

### Periodic acid schiff (PAS) stain

Periodic acid schiff [(PAS) (Sigma Aldrich, USA)] staining was performed for the estimation of glycogen storage levels in sections from all animal groups 30 days after MSCs and IL-6 treatment. After hydrating, paraffin sections were incubated in periodic acid for 5 minutes followed by staining with Schiffs reagent for 15 minutes and finally hematoxylin staining. Sections from three animals for each group and three sections per treatment were analyzed.

### Sirius red

Sirius red staining provided assessment of the collagen deposition in CCl_4_ injured and treated liver sections 30 days after MSCs and IL-6 treatment. Nuclei were stained with hemotoxylin (Sigma Aldrich, USA) and then picro-sirius red stain (Direct red 80, Sigma) was applied for 1 hour.

### Gene expression

Total RNA was extracted from injured and treated cells and also from liver tissues samples using TRIZOL (Invitrogen, USA). cDNA synthesis was carried out from 1 μg of RNA sample using M-MLV reverse transcriptase (Invitrogen, USA). For analysis of gene expression in MSCs after various treatments, real time RT-PCR was carried out using SYBR Green PCR Super Mix (BioRad, USA), 8 mM of each primer and 100–500 ng/ml of template cDNA on BioRad System iQ5. The relative ratio and standard deviation between the normal and treated samples were calculated using the comparative Ct method (DD Ct value), as recommended by the BioRad iQ5system. The expression of *Bax, Bcl-xl, albumin, caspase-3, cytokeratin-8, NFκB, TNF-α* was estimated by using Image J software. βactin was used as internal control. All primer sequences have been mentioned in Table 1.

**Table 1:** Primer sequences

**Gene**	**Sequence**	**Product size**
Bax (F)	TGGAGATGAACTGGACAGCA	182
Bax (R)	CAAAGTAGAAGAGGGCAACCAC	
Bcl-xl (F)	TTCGGGATGGAGTAAACTGG	150
Bcl-xl (R)	AAGGCTCTAGGTGGTCATTCAG	
Albumin (F)	GCTGTAGTGGATCCCTGGTG	196
Albumin (R)	GCTGTAGCCTTGGGCTTG	
Cytokeratin-8 (F)	CTCACTAGCCCTGGCTTCAG	232
Cytokeratin-8 (R)	ACAGCTGTCTCCCCGTGA	
Caspase3 (F)	TGTCATCTCGCTCTGGTACG	220
Caspase 3 (R)	AAATGACCCCTTCATCACCA	
NF-κB (F)	GCACCTGTTCCAAAGAGCAC	200
NF-κB (R)	GTGGAGTGAGACATGGACACAC	
TNF-α (F)	ACGGCATGGATCTCAAAGAC	162
TNF-α (R)	GGAGGTTGACTTTCTCCTGGTA	
βactin (F)	GCTGTGTTGTCCCTGTATGC	106
βactin (R)	GAGCGCGTAACCCTCATAGA	

### Biochemical tests

Blood samples were taken from all experimental groups 30 days after treatment with MSCs and IL-6. Serum was isolated and the amount of bilirubin (Diazyme Europe, Gmbh) and alkaline phosphatase (Bioassay System, USA) was estimated using commercial kits according to the manufacturer’s protocol.

### Statistical analysis

Quantitative data was obtained from 3 slides from each animal in different experimental groups for Sirius red staining and were expressed as means ± SEM. Analysis for percentage of fibrosis between groups was performed by one-way ANOVA (p value of less than 0.05 was considered statistically significant) with bonferroni post-hoc test. Data from10 animals/group was analyzed for bilirubin and ALP.

## Results

### In vitro hepatocyte injury model

**Figure 1:**
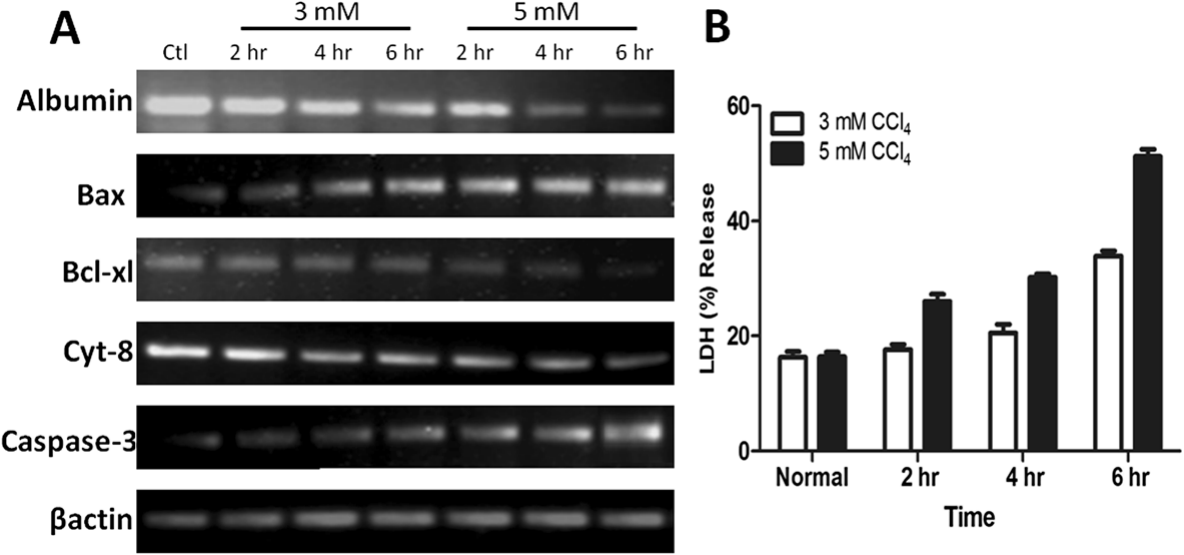
**In vitro hepatocyte injury model. A**) Gene expression analysis of hepatic (*Albumin, Cytokeratin-8*), apoptotic (*Bax, caspase-3*) and anti-apoptotic (*Bcl-xl)* markers after 3 mM and 5 mM CCl_4_ treatment is dose and time dependent as measured by RT-PCR (n=3). **B**) LDH (%) release in hepatocytes shows maximum release after 5 mM CCl_4_ treatment for 6 hours (n=3).

Hepatocytes were exposed to different concentrations of CCl_4_ i.e. 3 mM and 5 mM in medium for 2, 4 and 6 hours. Expression of apoptotic markers such as *Bax*, and *caspase-3* was significantly upregulated in 6 hours group while *Bcl-xl* was downregulated as measured by RT-PCR. Similarly, hepatic markers *albumin* and *cytokeratin-8* were decreased with increase in CCl_4_ concentration and duration of treatment (Figure 1A). Concurrently, level of cytotoxicity as measured by LDH assay was significantly higher 6 hours after 5 mM CCl_4_ treatment (Figure 1B). Therefore, 5 mM CCl_4_ concentration for 6 hours was used in subsequent experiments with in vitro cultured hepatocytes.

### Enhanced hepatocyte protection in vitro after MSCs + IL-6 treatment

**Figure 2:**
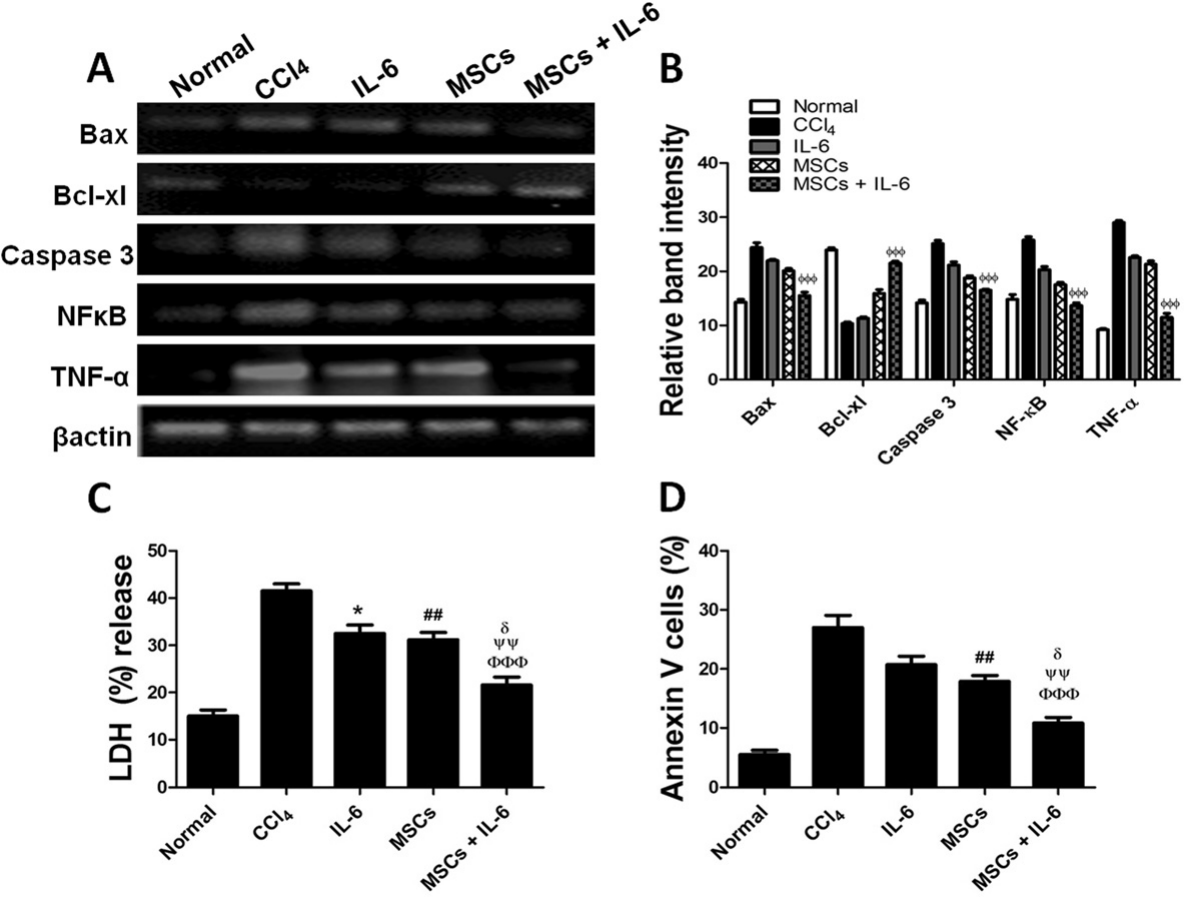
**Enhanced hepatocyte survival after co-culture with MSCs + IL-6. A**) Gene expression of apoptotic (*Bax, caspase-3, NF-κB, TNF-α*) and anti-apoptotic (*Bcl-xl*) markers in hepatocytes co-cultured with MSCs together with IL-6. **B**) Gel band quantification of gene expressions through Image J (n=3). CCl_4_ vs MSCs + IL-6 ^ɸ ^*p*< 0.05, ^ɸ ɸ ^*p*< 0.01 and ^ɸ ɸ ɸ ^*p*< 0.001. **C**) LDH (%) release levels in control and treatment groups (n=4). **D**) Analysis of apoptotic cells through annexin V staining (n=4). CCl_4_ vs IL-6 *p< 0.05, **p< 0.01 and ***p< 0.001, CCl_4_ vs MSCs ^#^*p*< 0.05, ^##^*p*< 0.01 and ^###^*p*< 0.001, CCl_4_ vs MSCs + IL-6 ^ɸ ^*p*< 0.05, ^ɸ ɸ ^*p*< 0.01 and ^ɸ ɸ ɸ ^*p*< 0.001, IL-6 vs MSCs + IL-6 ^ψ^*p*< 0.05, ^ψψ^*p*< 0.01 and ^ψψψ^*p*< 0.001, MSCs vs MSCs + IL-6 ^δ^*p*< 0.05, ^δδ^*p*< 0.01 and ^δδδ^*p*< 0.001.

Hepatocytes were subjected to CCl_4_ treatment (5 mM for 6 hours) followed by treatment with IL-6 for 24 hours. At the end of IL-6 treatment, MSCs were co-cultured with hepatocytes in a transwell system with or without IL-6. Hepatocyte co-cultures were divided into five groups i.e. Non-treated, CCl_4_, IL-6, MSCs and MSCs + IL-6 treated hepatocytes. Analysis of gene expression showed reduction in apoptotic markers such as *Bax, Caspase-3, NFκB* and *TNF-α* in hepatocytes treated with MSCs and IL-6 alone compared to CCl_4_ group but a significant reduction was observed after treatment with MSCs + IL-6 compared to all other groups. Similarly, increased levels of anti apoptotic marker *Bcl-xl* was observed after MSCs and IL-6 treatment alone but a greater reduction was observed after combined treatment of MSCs + IL-6 compared to CCl_4_ treated hepatocytes (Figure 2A-B). Significant reduction in LDH levels was observed in IL-6 (32.5 ± 1.8%) and MSCs (31.2 ± 1.5%) treated injured hepatocytes (Figure 2C). However, treatment with MSCs + IL-6 showed significant higher reduction (21.6 ± 1.6%) in cell injury than any other treatment groups. Conversely, level of apoptosis as measured by Annexin-V staining was lower in IL-6 (20.7 ± 1.4%) and MSCs (17.9 ± 0.9%) compared to CCl_4_ (27.0 ± 2.0%). However, MSCs + IL-6 treatment (10.8 ± 0.9%) showed significant reduction in Annexin-V staining compared to all other groups (Figure 2D).

### Homing of transplanted MSCs

**Figure 3:**
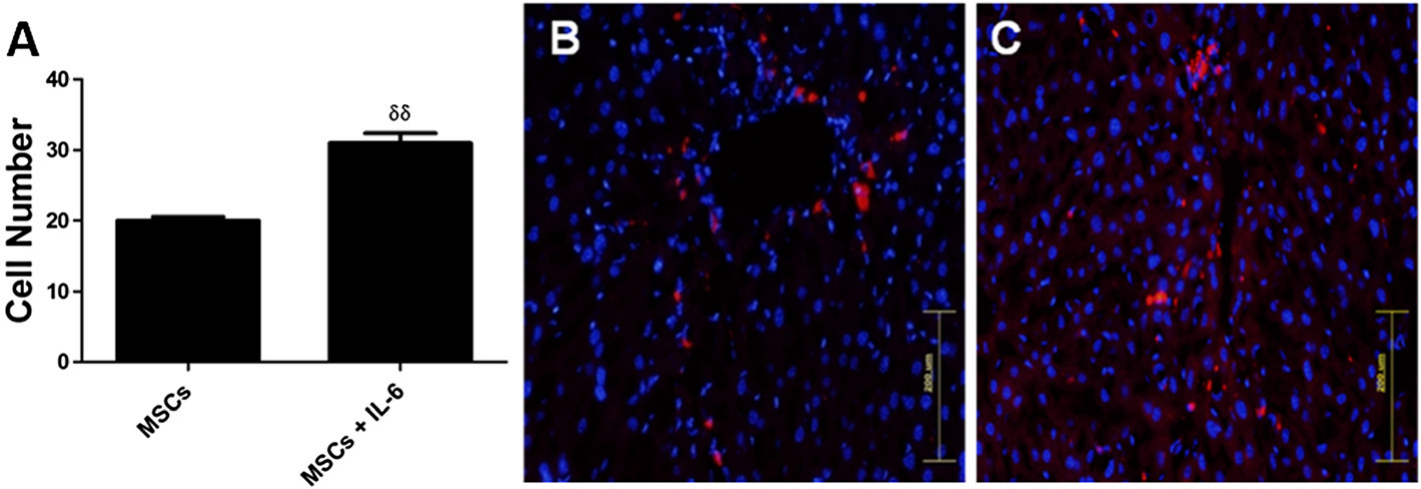
**MSC homing in injured liver 30 days post transplantation. A**) Quantification of transplanted MSCs (n=6). **B**) Injured liver with MSCs Transplantation. **C**) Injured liver with combined treatment (MSCs + IL-6). MSCs vs MSCs + IL-6 *p< 0.05, **p< 0.01 and ***p< 0.001.

MSCs labeled with PKH 26 and DAPI were transplanted into fibrotic livers in the presence or absence of IL-6. Increased homing in fibrotic liver from MSCs + IL-6 (Figure 3A-C) treatment group was observed as evidenced by increased number of PKH-26/DAPI colabeled cells (31 ± 3.2 cells/mm^2^) compared to MSCs (20 ± 1.2 cells/mm^2^) only transplantation group thus showing augmentation of MSC homing and engraftment in fibrotic livers treated with IL-6.

### Reduced fibrosis and apoptosis in CCl_4_ injured liver after treatment with MSCs + IL-6

**Figure 4:**
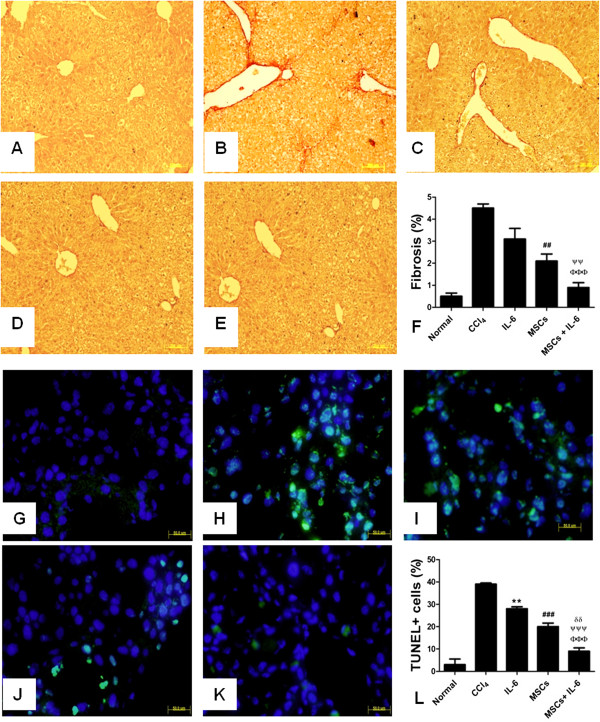
**Reduced fibrosis and apoptosis in injured liver treated with MSCs + IL-6. A**: Normal, **B**: CCl_4_ injury, **C**: IL-6 treated, **D**: MSCs transplanted, **E**: IL-6 + MSCs transplanted, **F**: Graphical presentation of collagen levels (n=). CCl_4_ vs IL-6 NS, CCl_4_ vs MSCs ^#^*p*< 0.05, ^##^*p*< 0.01 and ^###^*p*< 0.001, CCl_4_ vs MSCs + IL-6 ^ɸ ^*p*< 0.05, ^ɸ ɸ ^*p*< 0.01 and ^ɸ ɸ ɸ ^*p*< 0.001, IL-6 vs MSCs + IL-6 ^ψ^*p*< 0.05, ^ψψ^*p*< 0.01 and ^ψψψ^*p*< 0.001, MSCs vs MSCs + IL-6 ^δ^*p*< 0.05, ^δδ^*p*< 0.01 and ^δδδ^*p*< 0.001. G-L Estimation of apoptosis in control fibrotic liver and treatment groups through TUNEL Assay. **G**: Normal, **H**: CCl_4_ injury, **I**: IL-6 treated, **J**: MSCs transplanted, **K**: IL-6 + MSCs transplanted, **L**: Graphical presentation of apoptotic positive cells in different treatment groups (n=). CCl_4_ vs IL-6 *p< 0.05, **p< 0.01 and ***p< 0.001, CCl_4_ vs MSCs ^#^*p*< 0.05, ^##^*p*< 0.01 and ^###^*p*< 0.001, CCl_4_ vs MSCs + IL-6 ^ɸ ^*p*< 0.05, ^ɸ ɸ ^*p*< 0.01 and ^ɸ ɸ ɸ ^*p*< 0.001, IL-6 vs MSCs + IL-6 ^ψ^*p*< 0.05, ^ψψ^*p*< 0.01 and ^ψψψ^*p*< 0.001, MSCs vs MSCs + IL-6 ^δ^*p*< 0.05, ^δδ^*p*< 0.01 and ^δδδ^*p*< 0.001.

In order to demonstrate the combined treatment effect of MSCs and IL-6 on fibrosis and apoptosis in fibrotic liver, liver section from all treatment groups were stained with Sirius red for fibrosis and TUNEL staining for apoptosis detection. Mice treated with IL-6 and MSCs alone showed reduction in fibrosis which was 3.1 ± 0.4% and 2.1 ± 0.3% respectively compared to CCl_4_ treated animals (4.5 ± 0.1%) as evidenced by sirius red staining (Figure 4A-F). However, significant reduction in fibrosis was observed in MSCs + IL-6 treatment group (0.9 ± 0.2%) compared to all other groups. Conversely, TUNEL+ cells were reduced in fibrotic liver after IL-6 (28 ± 0.9%) and MSCs (20 ± 1.6%) compared to CCl_4_ (39 ± 0.5%) treatments (Figure 4G-L). However, there was significant reduction in TUNEL+ cells in MSCs + IL-6 (9 ± 1.5%) group compared to all other groups.

### Improved hepatocyte survival and function in vivo after MSCs + IL-6 treatment

**Figure 5:**
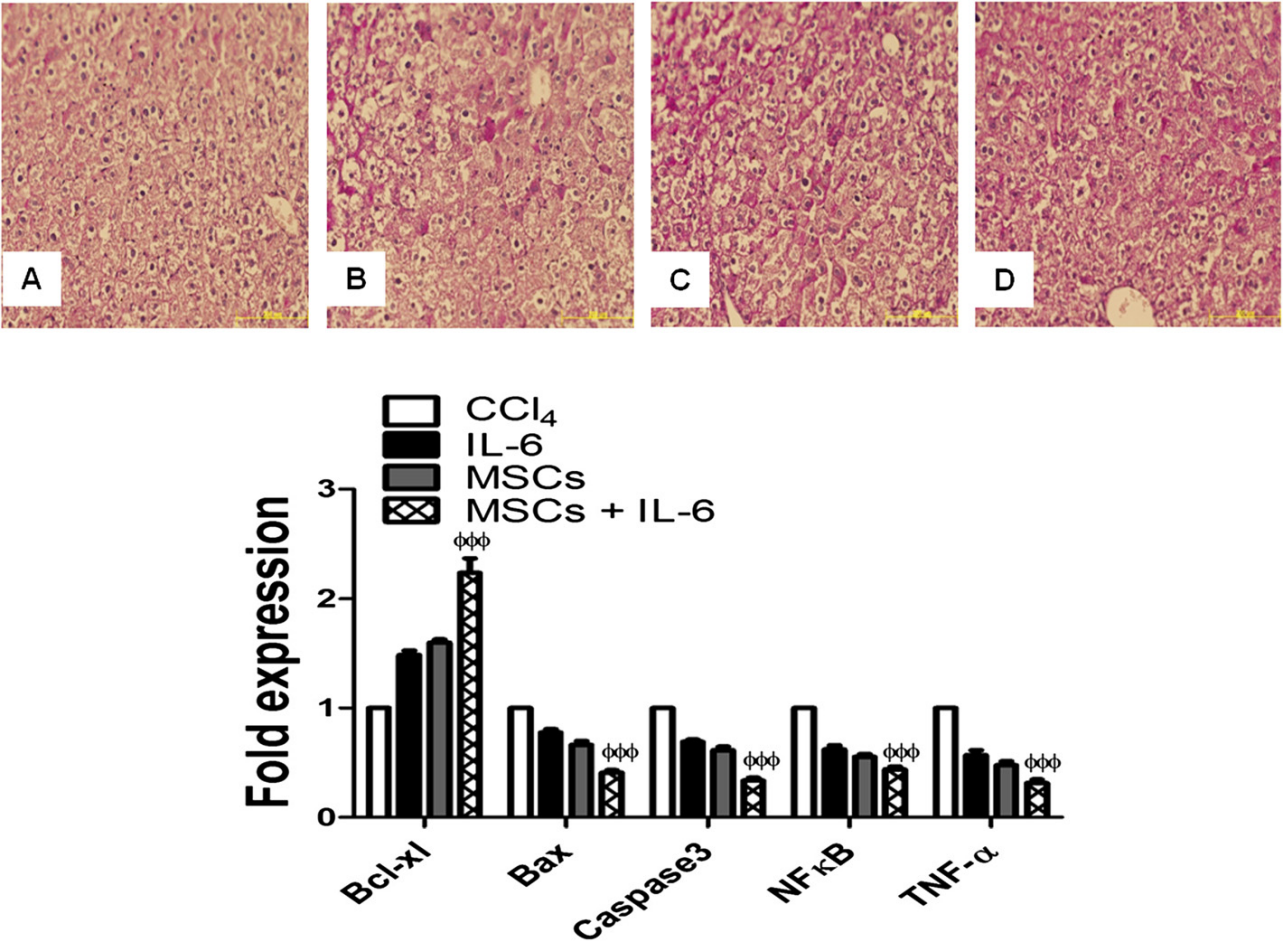
**Increased glycogen storage and survival signaling in livers after treatment with MSCs + IL-6.** PAS staining of liver section from **A**) - CCl_4_, **B**) IL-6, **C**) MSCs, **D**) MSCs + IL-6 treated animals (n=). **E**) Gene expression analysis of *bcl-xl, bax, caspase-3, NFκB, TNF-α* as measured by quantitative RT-PCR (n=). CCl_4_ vs MSCs + IL-6 *p< 0.05, **p< 0.01 and ***p< 0.001.

PAS staining of liver sections from all animal groups was done to evaluate the effect of combined treatment of MSCs and IL-6 on restoration of functional hepatocytes in liver damaged by CCl_4_ treatment. It was demonstrated that animals treated with MSCs + IL-6 showed high levels of glycogen storage compared to MSCs, IL-6 and CCl_4_ treated groups as evidence by PAS staining (Figure 5A-D). In addition, increased expression of *Bcl-xl* was observed in livers treated with MSCs + IL-6 compared to groups treated with either MSCs or IL-6 and CCl_4_ (Figure 5F). Similarly, expression levels of apoptotic markers such as *Bax, caspase-3, NF-κB and TNF-α* demonstrated significant reduction after combined MSCs + IL-6 administration compared both treatment alone and CCl_4_ treated groups (Figure 5E) providing evidence that MSCs + IL-6 administration enhances glycogen storing ability of hepatocytes, promotes survival and decreases apoptotic signaling.

### Biochemical functions

**Figure 6:**
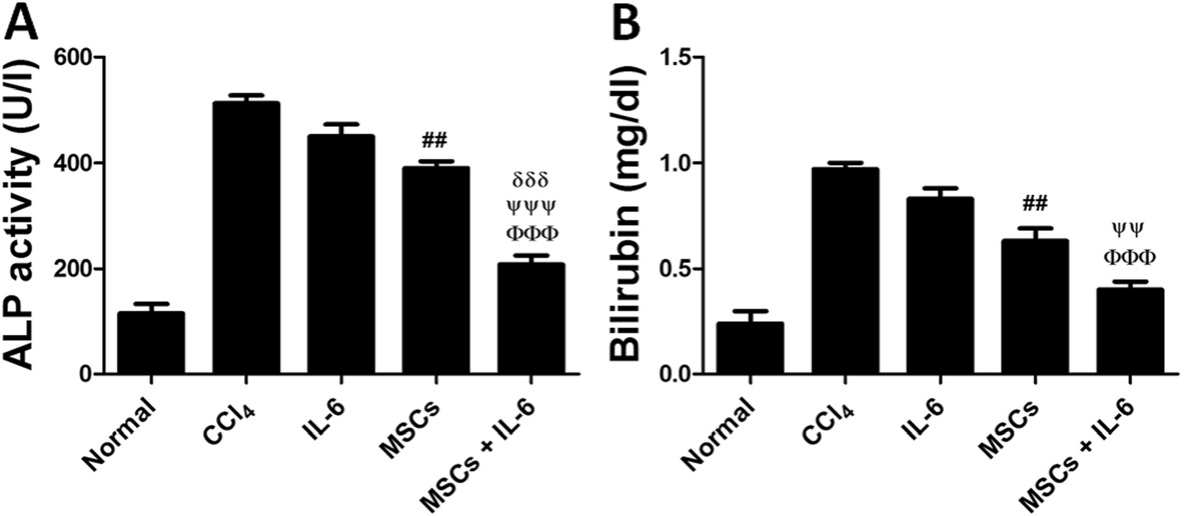
**Liver function tests. A**) Alkaline phosphatase in serum of all animal groups **B**) Bilirubin serum levels in all animal groups (n= 10/group). CCl_4_ vs IL-6 *p< 0.05, **p< 0.01 and ***p< 0.001, CCl_4_ vs MSCs ^#^*p*< 0.05, ^##^*p*< 0.01 and ^###^*p*< 0.001, CCl_4_ vs MSCs + IL-6 ^ɸ ^*p*< 0.05, ^ɸ ɸ ^*p*< 0.01 and ^ɸ ɸ ɸ ^*p*< 0.001, IL-6 vs MSCs + IL-6 ^ψ^*p*< 0.05, ^ψψ^*p*< 0.01 and ^ψψψ^*p*< 0.001, MSCs vs MSCs + IL-6 ^δ^*p*< 0.05, ^δδ^*p*< 0.01 and ^δδδ^*p*< 0.001.

To further evaluate the role of combined treatment with MSCs and IL-6 on augmentation of liver function, serum levels of Alkaline phosphatase (ALP) and Bilirubin were analyzed in all treatment groups. Serum levels of ALP were significantly lower in mice receiving combined treatment with MSCs + IL-6 (208 units/L) compared to treatment with IL-6 (450 units/L), MSCs (390 units/L) and CCl_4_ (613 units/L) groups (Figure 6A). Similarly, Bilirubin level in MSCs + IL-6 treated group was 0.4 mg/dl, which was significantly lower than IL-6 (0.83 mg/dl), MSCs (0.63 mg/dl) and CCl_4_ (1.17 mg/dl) treatment groups (Figure 6B). Collectively, these results indicate that combined treatment with MSCs and IL-6 results in higher recovery of hepatic function than either of the treatment alone compared to CCl_4_ treated mice.

## Discussion

Liver fibrosis is a progressive disease that involves disruption of hepatic tissue architecture and accumulation of extracellular matrix in response to pathological insults. Hepatocyte apoptosis as a consequence of prolonged liver disease is one of the hallmarks of liver fibrosis and leads to development of a fibrotic scar [24]. Bone marrow derived mesenchymal stem cells (MSCs) have been shown to develop into functional hepatocytes in vitro [25] and in vivo [26] proclaiming them as possible source for repair of damaged liver. However, recent reports indicate poor survival and engraftment of MSCs in hostile hepatic milieu [15,27]. There is a dire need of strategies that protect hepatocytes against injury promoting liver microenvironment for MSCs engraftment and survival. Therefore, in the present study we combined MSC transplantation for repair of fibrotic liver with IL-6 treatment with the premise that IL-6 treatment will protect hepatocytes from fibrotic injury thereby priming MSCs engraftment and survival.

A large number of studies have been conducted aimed at protecting hepatocytes from pathological injury thereby augmenting hepatic milieu and promoting repair. Hepatocyte protection from hypoxic injury was recently demonstrated by treatment with methylene blue and epimorphin in rats [28,29]. Similarly, treatment with different cytoprotective chemicals can protect hepatocytes against toxic injury [30-32]. Alternatively, treatment with cytokines released by hepatocytes in response to injury can enhance hepatocyte survival by activation of pro survival cascades. Preconditioning hepatocytes with IL-6, a major regulator of hepatic regeneration, before induction of injury promotes proliferation [13,33,34] and has a protective effect against apoptosis [11], ethanol and TNF induced injuries [35]. Conversely, IL-6 −/− mice display delayed weight recovery, defective DNA synthesis post operative mortality and severe liver fibrosis [36-38]. Exogenous administration of IL-6 in IL-6 −/− mice enhanced DNA replication to the levels seen in wild type mice [13,34]. Therefore, we sought to augment fibrotic liver tissue with exogenous IL-6 treatment with the premise that IL-6 administration will protect existing hepatocytes from CCl_4_ induced injury. Increased hepatocyte survival will prime the hepatic milieu for MSCs engraftment and survival leading to increase hepatic repair.

Recent evidence shows that soluble factors produced by bone marrow cells can functionally enhance hepatocytes in a co-culture system [21,39]. Conversely, IL-6 pretreated hepatocytes were co-cultured with MSCs to determine whether combined treatment with IL-6 and MSCs can protect hepatocytes against CCl_4_ injury. Increased survival was observed in hepatocytes pretreated with IL-6 co-cultured with MSCs in response to CCl_4_ induced injury as evidenced by decreased expression of pro apoptotic markers (*BAX, Caspase-3, TNF-α NFκB)* and LDH activity (Figure 2). Interestingly, co-culture of MSCs alone or IL-6 treatment did not result in a significant increase in hepatocyte survival compared to the combination of MSC co-culture with IL-6 treatment. Therefore, combination of IL-6 and soluble factors released by MSCs can result in significantly improved hepatocyte survival against CCl_4_ induced fibrosis than any other treatment modality.

Beneficial effect of combined MSC and IL-6 treatment on hepatocyte survival in vitro was extended in vivo to demonstrate that IL-6 can enhance hepatocyte survival and improve hepatic milieu that would lead to increased efficacy of MSC therapy for the repair of fibrotic liver. Recent studies show that IL-6−/− mice demonstrate hepatocyte apoptosis after treatment with ethanol emphasizing the role of IL-6 in liver protection against drug induced hepatoxicity [35]. Moreover, IL-6 can exert an antioxidant effect on hepatocytes by imparting protection against ROS while *Bcl2* and *Bcl-xl* levels were shown to increase in IL-6 dependent manner [11]. Improvement in hepatic environment by virtue of increased hepatocyte protection by IL-6 administration against CCl_4_ induced injury would enable enhanced MSC survival and hepatic repair. Concurrently, increased MSC homing was observed in IL-6 treated fibrotic livers compared to non-treated hepatic tissue (Figure 3) and was associated with reduced fibrosis and apoptosis (Figure 4). In addition, combined treatment of MSCs and IL-6 resulted in increased glycogen storing ability of livers affected by CCl_4_ induced fibrosis in conjunction with increased expression of *Bcl-xl* and decreased expression of *Bax, caspase-3, TNF-α* and *NFκB* (Figure 5). Augmented hepatic environment evidenced by increased hepatocyte survival leads to increased MSCs survival that significantly improved hepatic function demonstrated by serum levels of ALP and Bilirubin (Figure 6).

Cell therapy remains an attractive therapeutic modality for the treatment of liver fibrosis however, the efficacy of bone marrow derived MSCs within the hostile liver tissue remains a serious concern. MSCs have the ability to promote hepatocyte survival via paracrine mediated effects [21,39] and can differentiate into functional hepatocytes. Therefore, augmenting MSC capability for liver repair by enhancing liver tissue environment by IL-6 administration can lead to increased MSC homing and engraftment. Increased MSC survival within fibrotic liver pretreated with IL-6 leads to functional hepatic improvement and may be a novel therapeutic strategy for the treatment of liver fibrosis.

## Conclusion

We have demonstrated that IL-6 administration can significantly enhance the ability of MSCs to repair liver after CCl_4_ induced fibrosis. IL-6 is one of the pleiotropic inflammatory cytokines produced by the liver in response to injury. Recent evidence shows that IL-6 can enhance hepatocyte survival while IL-6 depleted mice have enhanced liver fibrosis. Furthermore, studies demonstrating MSC therapy for repair of liver fibrosis has debatable results warranting a new strategy to enhance the potential of MSCs for repair of damaged liver. MSC transplantation in fibrotic livers treated together with IL-6 results increases MSC engraftment leading to improved hepatic function. Therefore, we report here a clinically viable therapeutic option combining IL-6 treatment with MSCs transplantation for the treatment of fibrotic liver.

## Competing interests

The authors have no financial conflicts of interest.

## Authors’ contributions

All authors have read and approved the manuscript. GAN and SM designed research; GAN, GA, SS performed research; GAN and SM analyzed data; and GAN, SM, MK, SNK and SR. wrote the paper.
